# Effect of Carrier Agents on Quality Parameters of Spray-Dried Encapsulated Diosgenin Powder and the Optimization of Process Parameters

**DOI:** 10.3390/foods12122330

**Published:** 2023-06-09

**Authors:** Prajya Arya, Pradyuman Kumar

**Affiliations:** Department of Food Engineering and Technology, Sant Longowal Institute of Engineering and Technology, Sangrur 148106, Punjab, India; prajyaarya27@gmail.com

**Keywords:** diosgenin, optimization, powder morphology, process parameters, spray drying

## Abstract

Fenugreek seeds are a rich source of bioactive compounds, such as diosgenin, which is one of the most crucial steroidal sapogenins emerging in the field with its spectacular health benefits. Plant-based diosgenin is bitter in taste and has remarkably low consumption levels, making it unable to fulfil the role of improving health benefits. Diosgenin is spray dried to mask bitterness and astringent flavors with two different wall materials, such as maltodextrin (MD) and whey protein concentrate (WPC), separately. The spray-drying condition of the selected optimization process was inlet air temperature (IAT 150–170 °C), feed flow rate (FFR 300–500 mL/h), and carrier agent concentration (CAC 10–20%). The optimization of the process variable was conducted for producing optimized encapsulated diosgenin powder (EDP) with both MD and WPC. The selected parameters, such as yield, encapsulation efficiency, moisture content, antioxidant activity, hygroscopicity, and solubility, are investigated in this current work. Based on the experimental results, the significant R^2^ values depict the model fitting to the responses. EDP revealed an optimization condition at 170 °C IAT, 500 mL/h FFR, and 20% CAC for MD and WPC. The highest responses were observed with WPC-EDP, such as yield at 82.25%, encapsulation efficiency at 88.60%, antioxidant activity at 53.95%, and hygroscopicity at 12.64%. MD-EDP revealed higher solubility at 96.64% and moisture content at 2.58%. EDP was studied using micrographs and diffractograms for the optimized samples, which revealed a smooth and dented surface with an amorphous nature for MD-EDP and WPC-EDP, respectively. EDP exhibited acceptable powder properties with regard to fulfilling the set purpose. EDP can be a better potential ingredient in different food matrices to act as a delivery vehicle for various health aliments.

## 1. Introduction

The increasing demand for the development of nutraceuticals has encouraged many researchers to search for various bioactive compounds in different food matrices. The richness of Indian spices is yet to be explored, attracting scientists to explore their matrices. Among the various Indian spices, fenugreek is gaining attention due to the presence of numerous bioactive compounds, such as diosgenin [1]. Diosgenin is a steroidal aglycone compound that is produced through alternations in the chemical synthetic pathway. It aids in the production of various sex hormones, such as progesterone, estrogen, and steroidal drugs on an industrial level [2]. The medicinal properties of diosgenin aids in the prevention and treatment of various metabolic disorders, such as cancer, diabetes, cardiovascular disorder, obesity, hyperglycemia, blood and bone disorders, vasodilating effect, melanogenesis, and many others [3]. Diosgenin can be extracted from different sources, such as *Trigonella foenum graecum* (fenugreek), *Dioscorea* spp. wild yam, and many more. The diosgenin was extracted from fenugreek using ultrasound-assisted extraction conducted by Arya and Kumar [4]. The extracted diosgenin has a bitter taste and astringent flavor that is unacceptable in terms of its sensory profile. The remarkable health properties of diosgenin have attracted the attention of researchers who seek to mask the bitter taste and astringent flavor of fenugreek-extracted diosgenin. The most likely and suitable way to make diosgenin more palatable is to extract it using a suitable technique. Spray drying is a suitable technique for encapsulating diosgenin [5].

Spray drying is the technique of converting a liquid feed into the spray-dried powder with the aid of a carrier agent that masks the core material and protects it against internal and external environmental factors, such as pH, temperature, digestive juice, and light. Spray drying aids in the good storage potential of sensitive bioactive compounds by covering them with a suitable drying agent and converting them in powder with a low moisture content. It also helps to occupy less storage space and has an extended shelf life [6]. The selection of suitable wall materials completes the encapsulation, along with the selection of process parameters. The wall materials aid in the completion of the spray-drying process by providing an encapsulating shield against the selected variables for the core material [7]. The spray drying of diosgenin was conducted using the two most-common wall materials: maltodextrin (MD) and whey protein concentrate (WPC) [8]. The variation in the characteristics of the powders depends on the chosen wall material. The usage of maltodextrin in spray-dried food powders results in a variety of powder products. MD is a complex polysaccharide that has a hygroscopic nature with better encapsulation efficiency and higher bulk density; however, it tends to be a sticky powder because of its low glass-transition temperature [9]. In contrast, WPC is a protein-complex wall material based on a protein matrix that facilitates rapid heat and mass transfer during drying, and results in leaving behind a powder with lower hygroscopicty and bulk density values and is less sticky [10]. There are many different types of spray-dried powder produced with MD, such as pomegranate extract [11], curcumin [12], garlic oleoresin [13], and WPCs, such as blueberry [14], peanut sprout [15], garcinia juice, and many more [16].

In view of the bitter taste of fenugreek seed-extracted diosgenin makes it less palatable due to the unacceptable flavor. The encapsulation uses both MD and WPC for masking the bitterness and astringent flavor of diosgenin. This research paper focuses on the effect of carrier agents MD and WPC, inlet air temperature, feed flow rate on yield, encapsulation efficiency, moisture content, antioxidant activity, hygroscopicity, solubility, and its optimization using the response surface methodology. The optimized encapsulated diosgenin powder (EDP) from both MD and WPC was evaluated for studying the powder characteristics, such as surface particle morphology and X-ray diffractograms.

## 2. Materials and Methods

### 2.1. Feed Preparation

The fenugreek seed-extracted bioactive compound diosgenin was selected for the encapsulation. Encapsulation was performed using two different wall materials: maltodextrin with dextrose equivalent 20 (MD) and whey protein concentrate with 80% concentrate (WPC). The chemicals, MD, and WPC used for the analysis of the spray-dried diosgenin powder were recovered from Loba Chemie and Mahaan Foods, India. The spray-drying feed was prepared by dissolving varying amounts of carrier agent concentrations, followed by other independent variables, such as feed flow rate and inlet air temperature selected for RSM. The diosgenin was added to the feed in limited amounts due to its steroidal nature. The liquid feed was fed to the spray drier via a feed pump through an atomizer which enters into the drying chamber. Inside the drying chamber, the liquid feed was converted into tiny, fine spray-dried droplets because of the interaction with high inlet air temperature. The spray-dried microparticle were collected from the cyclone of the spray dryer. The initial amount of fenugreek used for diosgenin extraction was 100 g of defatted fenugreek power. The diosgenin extracted with the UAE method ranged from 40 mg/100 g of defatted fenugreek seed powder [5]. The sample preparation was based on the previous work performed for fixing the trial amount of diosgenin that varied from 0.1 to 0.5%, and 0.3% was selected for the spray drying [17]. Based on the previous studies, 0.3% diosgenin was added to the spray-dried feed separately to produce encapsulated diosgenin powder with MD and WPC. The recommended diosgenin intake should not be more that 510 mg/kg/day with no significant toxicity [18]. The selected dependent variables for the encapsulation of diosgenin included inlet air temperature (IAT, 150–170 °C), feed flow rate (FFR, 300–500 mL/h), and carrier agent concentration (CAC, 10–20%), as shown in Table 1. The atomizer for the spray dryer had a 0.5 mm diameter, concurrent air flow, and a peristaltic pneumatic pump to regulate the feed flow rate. The feed was fed into a pilot-scale spray dryer (SM Scientech, Kolkata, India). The produced encapsulated diosgenin powder (EDP) from both wall materials (MD and WPC) was kept in an airtight container until further use.

### 2.2. Yield

The yield of EDP was calculated for each drying aid [19] using Equation (1):

(1) $$\mathrm{Powder}\mathrm{Yield}\left(\%\right)=\frac{\mathrm{Mass}\mathrm{of}\mathrm{powder}\mathrm{obtained}\left(g\right)}{\mathrm{Mass}\mathrm{of}\mathrm{dried}\mathrm{diosgenin}+\mathrm{Mass}\mathrm{of}\mathrm{MD}\mathrm{and}\mathrm{WPC}}\;*\;100\ldots$$

### 2.3. Microcapsule Characterization

#### 2.3.1. Encapsulation Efficiency (EE, %)

For the estimation of the encapsulation efficiency of EDP, 50 mg of powder was dissolved in 50 mL of distilled water for surface and total diosgenin values. The sample was vortexed for 5 min, followed by centrifugation at 4000× *g* for 5 min. The filtrate was collected for total diosgenin content, and residual broken particles were analyzed for surface diosgenin content by washing it with ethanol 3 times [20]. The total and surface diosgenin contents were estimated by dissolving 0.2 mL of each sample (MD-EDP and WPC-EDP), followed by the addition of two-color reagents CR_1_ and CR_2_ and kept in a water bath at 60 °C for 30 min. The 0.5 mL distilled water was added to the samples for the estimation of diosgenin content in MD-EDP and WPC-EDP by spectrophotometric observation recorded at 430 nm [21]. Encapsulation efficiency was estimated by using Equation (2):

(2) $$Encapsulation\;Efficiency\left(\%\right)=\frac{Total\;diosgenin-Surface\;diosgenin}{Total\;diosgenin}\;*\;100\ldots$$

#### 2.3.2. Moisture Content (MC, %)

EDP was analyzed for moisture content by placing 5 g of powder in a hot-air oven at 105 ± 5 °C for 5 h [19]. The moisture content was calculated using Equation (3):

(3) $$MC\;\left(\%\right)=\frac{Mass\;of\;powder\;before\;drying\left(g\right)-Mass\;of\;powder\;after\;drying\left(g\right)}{Mass\;of\;powder\;before\;drying\left(g\right)}\;*\;100\ldots$$

#### 2.3.3. Antioxidant Activity (AA,%)

The antioxidant activity of EDP was estimated by the DPPH method with some modifications. A total of 10 mg of MD-EDP and WPC-EDP each was dissolved in 1 mL of distilled water followed by dissolving 3.4 mg of DPPH in 100 mL methanol. 0.1 mL of diosgenin powder extract, and 3.9 mL of DPPH solution were mixed together. The sample was kept in the dark for 45 min and observance was recorded at 515 nm with methanol blank [22]. The free-radical scavenging capacity of EDP was calculated using Equation (4):

(4) $$DPPH\;Scavenging\left(\%\right)=1-\frac{Absorbance\;of\;sample}{Absorbance\;of\;control}\;*\;100\ldots$$

#### 2.3.4. Hygroscopicity (HG, %)

The hygroscopicity of the EDP was estimated by placing 1 g of powder in pre-weighed petri dish kept in a desiccator containing an ammonium sodium chloride solution with a relative humidity value of 80% [23]. The powder was kept for 14 days for hygroscopicity studies. The hygroscopicity was calculated using Equation (5):

(5) $$HG\left(\%\right)=\frac{Sample\;weight\;after\;storage\left(g\right)-Samplestorage\;before\;storage\left(g\right)}{Initial\;weight\;of\;sample}\;*\;100\ldots$$

#### 2.3.5. Solubility (SB, %)

EDP was analyzed for solubility by placing 0.1 g of powder in 10 mL of distilled water. The sample was vortexed and centrifuged at 3000 rpm for 10 min. The supernatant was transferred to a pre-weighed petri dish and dried at 105 °C for 3h. Solubility was calculated by using the weight difference between the original sample weight and supernatant dried in the pre-weighed petri dish [24].

#### 2.3.6. Powder Morphology

The EDP surface morphology was analyzed by using a scanning electron microscope (JSM-7610, F PLUS, JOEL Ltd., Tokyo, Japan). The samples were prepared using gold coating on the powder particles using focused ion beam etching. The micrographs of the MD-EDP and WPC-EDP were recorded from 500× to 3500×, respectively [25].

#### 2.3.7. X-ray Diffraction

EDP was analyzed for the estimation of the powder type by using X-ray diffractometer (D8 Advance, Bruker, Germany) using a Cu-based anode X-ray tube. The powder sample was kept against the glass slide in an aluminum holder and measurements were observed using a diffraction angle ranging from 10° to 80° (2θ) with a scanning rate of 4°/min at 30 mA and 40 kV [26].

### 2.4. Experimental Design

The experiments were conducted for the optimization of the process parameters with a central composited design (CCD). The encapsulated diosgenin powder was produced in twenty experimental runs performed with six center points, as shown in Table 1. Design expert software (Minneapolis, 11.0.4.1, MA, USA) was used for conducting the experiments, followed by the optimization of data and a quadratic model being used for expressing the response variable as a function of the independent variable. The accuracy of the model selected was analyzed by closely observing various parameters, such as ANOVA, R^2^, adjusted R^2^, lack of fit, and coefficient of variation (CV) [27]. The real and coded values of factors are presented in Table 1 for both the carrier agents MD and WPC. The statistical significance was analyzed for selected responses, such as yield, encapsulation efficiency, moisture content, antioxidant activity, hygroscopicity, and solubility. The equation below was used for the estimation of the factors and models used in the optimization:

$$Y=\beta_{o}+\sum_{i=1}^{K}\beta_{i}X_{i}+\sum_{i=1}^{K}\beta_{ii}X_{i}^{2}+\sum_{i=j}^{K}\sum_{j=i+1}^{K}\beta_{ij}X_{ij}+\varepsilon$$

where Y: the desired value of the response, such as Y1 = yield (%); Y2 = encapsulation efficiency (%); Y3 = moisture content (%); Y4 = antioxidant activity (%); Y5 = hygroscopicity (%); and Y6 = solubility (%). *xi* represents the coded independent variables (*x*1 = inlet air temperature, *x*2 = feed flow rate, and *x*3 = carrier agent concentration); *βo* is the constant; and *βi*, *βii*, and *βij* are the linear, quadratic, and cross-product coefficients, respectively, and ε is an error.

## 3. Results

### 3.1. Model Fitting

The impact of the independent variables IAT, FFR, and CAC (MD and WPC) were observed for the dependent variables, such as yield, encapsulation efficiency, moisture content, antioxidant activity, hygroscopicity, and solubility, as represented in Table 2. The responses revealed a confidence level of 95% with ANOVA and were coded for a second-order regression equation, as represented in Table 3. The dependent variables showed n acceptable values for R^2^ as observed with values for MD-EDP and WPC-EDP for yield (0.9973, 0.9961), encapsulation efficiency (0.9848, 0.9874), moisture content (0.9921, 0.9910), antioxidant activity (0.9830, 0.9955), hygroscopicity (0.9847, 0.9906), and solubility (0.9909, 0.9918), respectively. After erasing the non-significant terminologies from the model, significant data are presented in Table 3.

### 3.2. Yield

**Figure 1:**
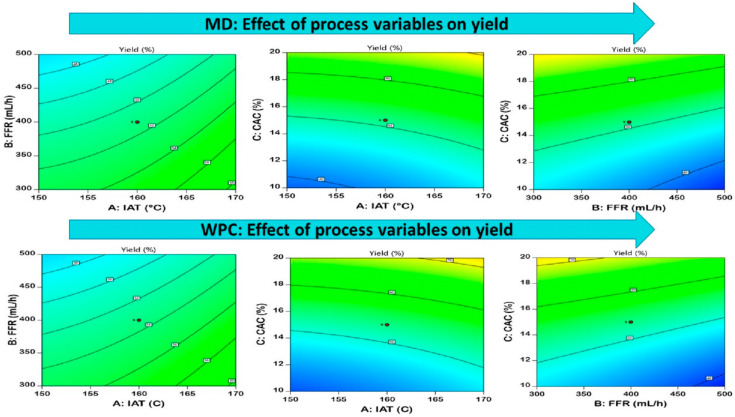
Pictorial representation of the contour plots generated for the encapsulated diosgenin powder yield with regard to maltodextrin and whey protein concentrate affected by spray-drying process parameters.

Encapsulated powder yield was one of the most crucial factors to be analyzed for estimating the efficacy of the spray-drying process [28]. The effect of spray-drying parameters was observed on the EDP yield that ranged from 34.12 to 80.65% and 36.76 to 82.25% for MD-EDP and WPC-EDP, respectively, as represented in Table 2. The analysis of variance for EDP yield, shown in Table 3, which depicted a significant fit for the quadratic model and a non-significant lack of fit. The impact of the selected independent variables IAT, FFR, and CAC was observed in favor of yield. The increase in IAT and FFR resulted in a considerable increase in the EDP yield, which was observed positively, whereas the impact of CAC had a moderate effect on the EDP yield with a medium-range-producing maximum yield with both the carrier agents MD and WPC. There was a lower yield observed in the case of MD as compared to WPC, due to the greater stickiness during the drying operation occurring in the drying wall chamber. The impact of CAC was observed in the EDP yield in the form of increasing an carrier agent, causing a higher yield with a significant effect (*p* < 0.05). The wall-forming capacity of the maltodextrin occurred up to certain limit due to the rapid heat and mass transfer that occurred during the drying process. The feed that contained MD as a wall material showed a quick response with regard to the moisture evaporation from the droplet surface during the drying of the atomized feed particles, and it revealed that rapid responses of MD during drying caused a lower yield. The contour plots of MD and WPC with impact values observed in the EDP are shown in Figure 1. The equation generated from the model optimization is represented in Table 4. A similar effect of MD was observed on the powder yield for the production of red–purple food colorant from *Opuntia stricta*, where the maximum yield ranged up to 54.63% [29]. The feed containing WPC as the carrier agent concentration revealed a better yield due to reduced adhesion. The higher yield in WPC was due to the greater interaction among protein and surface-active elements present on the periphery of the droplet. The surface-active elements aided in forming a film over the droplets that reduced the contact time between the dryer chamber wall and caused a potential reduction in stickiness and enhanced the powder yield [30]. A similar trend of results was observed for WPC spray-dried bayberry juice powder, with a yield ranging from 45.6 to 56.2% [31]. 

### 3.3. Powder Characterization

#### 3.3.1. Encapsulation Efficiency (EE)

**Figure 2:**
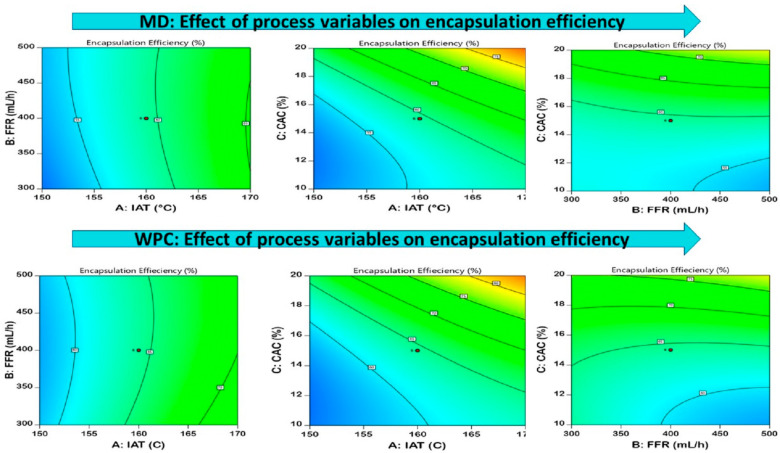
Pictorial representation of the contour plots generated for the encapsulated diosgenin powder encapsulation efficiency with regard to maltodextrin and whey protein concentrate affected by spray-drying process parameters.

Encapsulation is a physico-mechanical process comprising the entrapment of bioactive compounds in various sorts of carrier agents increasing the stability of the encapsulated compound. The encapsulation efficiency of spray-dried food powders majorly impacted efficient targeted delivery, safety from environmental changes, and efficacy in retaining bioactive compounds [32]. The encapsulation efficiencies for MD-EDP and WPC-EDP ranged from 50.49 to 83.33% and 51.27 to 88.60%, respectively. The contour plots of encapsulation efficiency are presented in Figure 2 for MD and WPC. The encapsulation efficiency was impacted by IAT and CAC with a positive impact and significant difference (*p* < 0.05), and FFR presented a negative impact, as shown in Table 3. A better EE value was observed with WPC in comparison with MD, due to the good surface protein interactions and binding with diosgenin. The increase in IAT and CAC caused an increase in EE by reducing the contact time to form a semi-permeable membrane crust over the droplet that caused suppressed interactions of diosgenin and the particle surface during drying [33]. A similar study conducted with regard to spray-dried nettle-extract powder ranged from 63.23 to 87.21% with an increase in IAT and MD concentrations [34]. The effect of WPC in regard of EE observed with flax seed oil microencapsulation ranged from 62.3 to 95.7%, revealing better EE values as compared to other wall materials [35]. The effect of spray drying was observed on the loading capacity of iron microcapsules prepared with glucomannan that ranged from 69.43 to 74.46% [36]. The equation generated from the model optimization is presented in Table 4.

#### 3.3.2. Moisture Content

**Figure 3:**
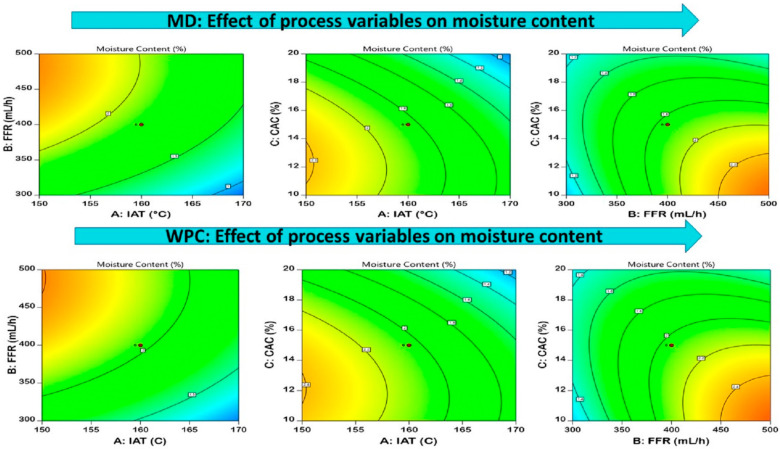
Pictorial representation of the contour plots generated for the encapsulated diosgenin powder moisture content with regard to maltodextrin and whey protein concentrate affected by spray-drying process parameters.

The moisture content of MD-EDP and WPC-EDP ranged from 0.65 to 2.58% and 0.82 to 2.76%, respectively. The effect of IAT was observed positively on the EDP with significant differences (*p* < 0.05), as the contour plot shows in Figure 3, for MD and WPC. The increase in IAT and CAC caused rapid moisture removal from the droplet surface and the moisture content decreased to an acceptable level [37]. A reduced level of moisture content was observed for MD-EDP due to the more and rapid interaction of the MD-feed atomized droplet with hot air inside the drier chamber. The retention of moisture content in the atomized droplet was a bit higher in WPC-EDP due to the greater interaction of feed moisture content and surface-active protein elements that bind with intermolecular spaces. The entrapment of higher moisture levels in WPC-EDP due to the dented-surface phenomenon occurred due to the hydrophilic nature of the whey protein concentrate as compared to MD [38]. A similar study performed for the spray drying of Sohiong fruit powder which had moisture content ranging from 3.15–4.51% [39]. The study was also conducted for the spray drying of tomato powder with different wall materials. The most suited range of moisture content was revealed for WPC, ranging from 3.41 to 4.71 g/100 g [40]. The moisture content of spray-dried powders was observed in the range of 2.89–4.12% for the stability of the food powders [41]. The equation generated from the model optimization is represented in Table 4.

#### 3.3.3. Antioxidant Activity

**Figure 4:**
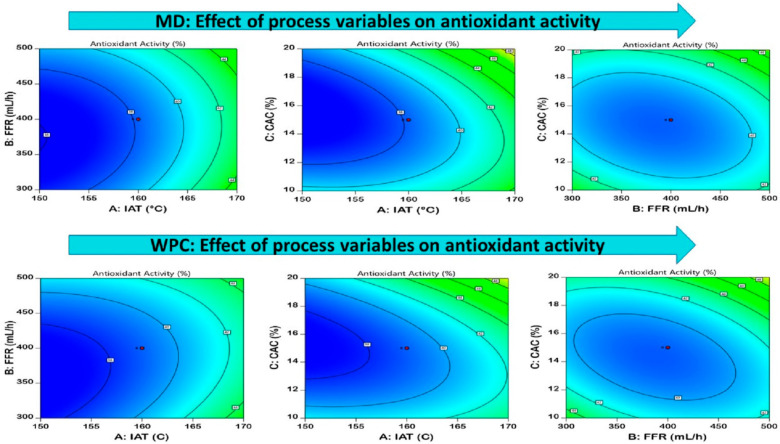
Pictorial representation of the contour plots generated for the encapsulated diosgenin powder antioxidant activity with regard to maltodextrin and whey protein concentrate affected by spray-drying process parameters.

Antioxidant activity is one major factor that counts for the bioactivity of spray-dried powders. The antioxidant activity of MD-EDP and WPC-EDP ranged from 37.01 to 52.71% and 37.34 to 53.95%, respectively, with the significant differences (*p* < 0.05) and contour plots shown in Figure 4 for MD and WPC. A certain decrease was observed in the samples whose drying was conducted at a higher spray-drying inlet air temperature causing a reduction in the antioxidant activity. This decrease was observed due to the reduced stability of antioxidants against higher processing temperature conditions. The influence of WPC was observed in the form of a film formation on the atomized feed droplets that prevented the degradation of the antioxidants, as compared with the MD with regard to EDP. The significant effect of IAT observed on EDP with an increase in IAT caused a reduction in antioxidant activity. Better antioxidant activity was observed in the case of WPC-EDP, rather than MD-EDP, due to the increased emulsifying properties and gelling capacity that aided the antioxidant activity [14]. A similar effect was observed for spray-dried amla powder produced with MD as a carrier agent, revealing its antioxidant activity. A reduction was observed in amla powder antioxidant activity with a higher concentration of MD due to an increased entrapment of the bioactive compound of amla inside the droplet during the drying process [42]. The effect of MD as a drying carrier agent was observed for antioxidant activity in black garlic aqueous extract powder. A reduction in antioxidant activity was revealed in black garlic powder with an increase in MD concentration [43]. A study was conducted for cookies prepared from the spray drying of blackcurrant concentrate with whey protein isolate. A better retention of the antioxidant capacity was observed with a higher radical scavenging capacity in the cookies [44]. The antioxidant activity of beetroot juice powder produced with WPC using the spray-drying technique ranged from 68.85 to 77.29% [45]. The equation generated from the model optimization is presented in Table 4.

#### 3.3.4. Hygroscopicity

**Figure 5:**
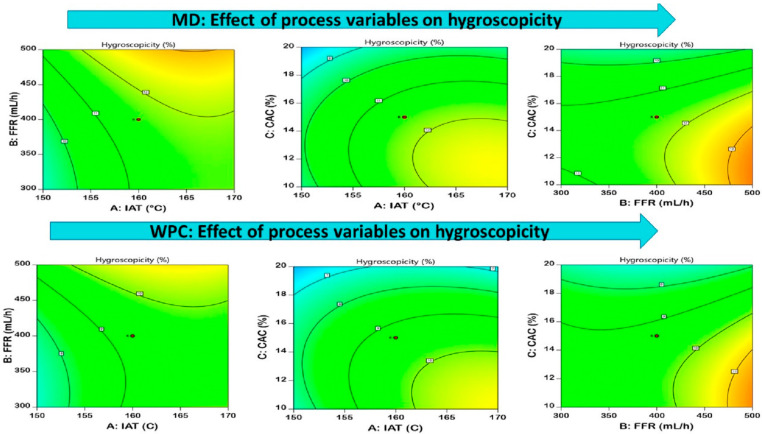
Pictorial representation of the contour plots generated for the encapsulated diosgenin powder hygroscopicity with regard to maltodextrin and whey protein concentrate affected by spray-drying process parameters.

Hygroscopicity is the crucial property of spray-dried powder that depicts the moisture hold-up and shelf-life properties of the powder [46]. The hygroscopicity of MD-EDP and WPC-EDP ranged from 6.64 to 13.52% and 4.82 to 12.64%, respectively, with significant differences (*p* < 0.05) as contour plots shown in Figure 5 for MD and WPC. The effects of IAT and CAC were observed on the hygroscopicity of EDP. MD-EDP had greater hygroscopicity as compared to WPC-EDP due to the higher molecular weight of the carrier agent causing a reduction in hygroscopicity, hence causing an overall reduction in the moisture absorption rate of the spray-dried powder [25]. The higher IAT caused a greater reduction in the hygroscopicity of the spray-dried powders. The feed that was spray dried at lower temperatures tended to absorb a higher moisture content and increase hygroscopicity. The low IAT dried powder produced a higher moisture content and reduced glass-transition temperatures that caused greater hygroscopicity of the powder [23]. The use of MD could modify the hydrophilic and hydrophobic interaction of the sprayed feed particles and helped to suppress the amount of adsorbed water [47]. The effect of MD was recorded in the form of higher MD concentration, reducing the hygroscopicity of the powder. The presence of hydrophilic and hydrophobic bonds and their interaction between wall and core materials caused a reduction in the amount of water absorbed by the droplet [48]. A similar study conducted for the spray-dried cempedak fruit powder revealed hygroscopicity that ranged from 30–38%. The impact of inlet air temperature and carrier agent concretion with MD showed a significant difference for hygroscopicity [49]. A similar trend for hygroscopicity was observed for tamarind pulp powder that ranged from 16.61 to 28.96% with WPC. The equation generated from the model optimization is presented in Table 4.

#### 3.3.5. Solubility

**Figure 6:**
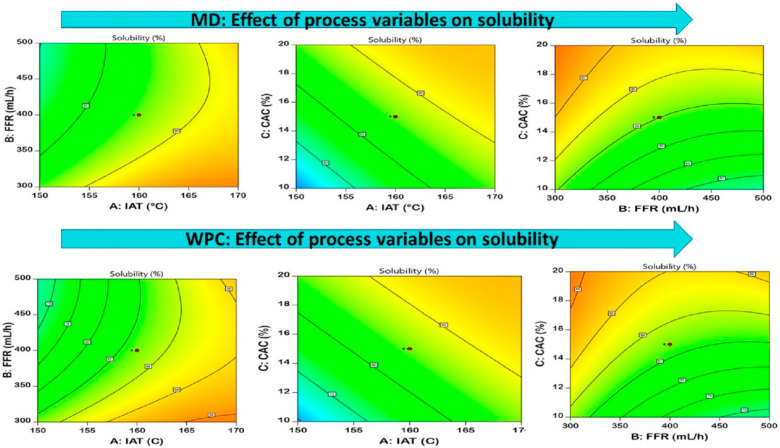
Pictorial representation of the contour plots generated for the encapsulated diosgenin powder solubility with regard to maltodextrin and whey protein concentrate affected by spray-drying process parameters.

The concept of evaluating the solubility of spray-dried powder depended on the criteria of the product’s behavior in aqueous phase and was majorly considered as the final step toward dissolution and reconstitution quality [50]. The solubility values of MD-EDP and WPC-EDP ranged from 75.84 to 96.64% and 70.92 to 91.72%, respectively, as represented in Figure 6, with a contour plot revealing a significant difference. Better solubility was observed in MD-EDP as compared to WPC-EDP due to the greater solubility of MD as compared to WPC in solvents [51]. The spray drying of sumac extract encapsulated with maltodextrin, conducted for studying the solubility levels, revealed that less time was consumed for the dissolution of the spray-dried powder [52]. The solubility of soybean hydrolysates was observed more with MD as compared to WPC, ranging from 94 to 97% with spray drying [51]. A study conducted for the spray drying of jujube extract using whey protein as a carrier agent presented lower solubility of up to 98%, as compared to sodium alginate [53]. The equation generated from the model optimization is presented in Table 4.

### 3.4. Optimizations of Carrier Agent Concentration, IAT, and FFR

The optimizations of the MD-EDP and WPC-EDP were performed using two different carrier agents, and their impactful changes were observed in the upper and lower limits followed by the predicted and experimental values during the optimization for the selected parameters, as shown in Table 5. The encapsulated diosgenin powder was evaluated based on the sensory acceptance that predicted the scores of 7.5 and 8 out of a 9-point hedonic scale for MD-EDP and WPC-EDP, respectively, which showed the masking of the bitter taste of diosgenin by the encapsulation process.

#### 3.4.1. Powder Morphology

**Figure 7:**
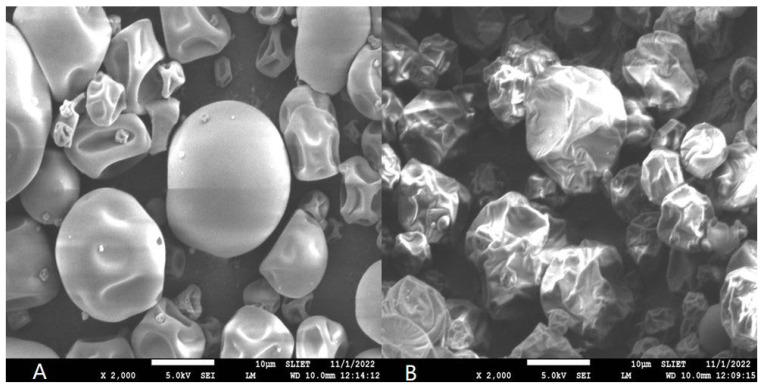
Pictorial representations of the micrographs of MD-EDP (**A**) and WPC-EDP (**B**) for the optimized samples at 2000×.

The morphology of spray-dried powder revealed the superficial structure of the microcapsules impacted due to process parameters. The most commonly occurring shapes were spherical, dented, clubbed, and shrunken, depending on the type of carrier agent, its concentration, and other process parameters [25]. Followed by the optimization of EDP, the most-suited samples were analyzed for the microstructural analysis by scanning electron microscope. The micrographs of optimized MD-EDP (A) and WPC-EDP (B) are presented in Figure 7 at a 2000× magnification as A and B, respectively. MD-EDP had more round-shaped, spherical microparticles, revealing the effect of the MD as a drying carrier agent. The round particles are shown with medium interparticle adhesion. The increasing concentration of MD resulted in the higher viscosity of the feed and formed a gel-like layer over the feed-atomized droplets that produced rounder particles. A similar effect was observed in the apple juice concentrate spray dried with MD as a carrier agent [28]. WPC-EDP had more wrinkled and dented surface microparticles, revealing the interaction of WPC during drying. The dented shape of the microparticles occurred due to irregular droplet shrinkage during the initial phase of drying. The moisture removal that occurred during drying caused the rapid formation of an interface that took place among the drying air, types of drying agent used in the feed, and droplet surface. The WPC produced more irregular-shaped microparticles as studied during the production of iron–WPC complex containing microparticles [10]. The chemical formulation of maltodextrin and whey protein concentrate altered the surface structure of spray-dried particles. The interaction of starches (maltodextrin) and protein (whey protein concentrate) during spray drying cause smooth round- and dented-ball effects, respectively. The starches generally presented a smooth walled effect due to the strong crosslinking of the molecular forces that did not alter the surface structure much. The proteins produced dented surfaces due to the presence of ionic groups in the feed particles that created obstructions during heat and mass exchange of the feed particles. A similar effect was observed for microparticles produced with WPC at different concentrations, revealing wrinkled and hollow structures produced using spray-drying technology [54].

#### 3.4.2. X-ray Diffraction

**Figure 8:**
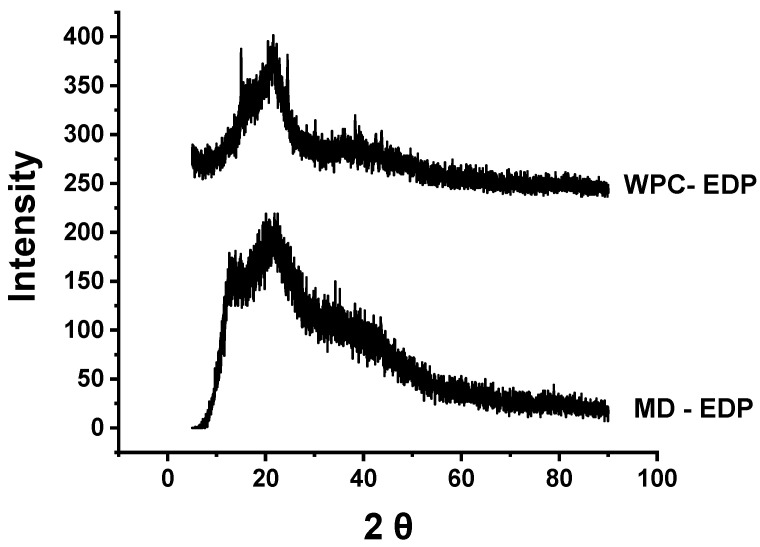
Representation of MD-EDP and WPC-EDP for X-ray diffractograms.

The interaction of diosgenin with MD and WPC produced an amorphous spray-dried powder. The effect of carrier agents was observed for the optimized samples obtained from MD-EDP and WPC-EDP, as shown in Figure 8. EDP depicted the relevant broad peaks and noise produced in the diffractograms. The XRD pattern of MD-EDP presented broader peaks, as compared to WPC-EDP. There was no considerable difference among the diffractograms obtained from MD and WPC. MD-EDP had a slightly greater crystallinity, because of the broader peak, due to the higher moisture absorption rate of the carrier agent. WPC-EDP had a less-broad peak due to the greater amorphous nature and reduced crystallinity revealed by the diffractograms. The less-amorphous powder had higher solubility that caused handling difficulty and may have produced crystallinity during storage; the higher amorphous nature of the powder prevented the release of the encapsulated substance from the matrix [55]. The produced microcapsules had very little crystallinity that is generally accepted for spray-dried food powders. The occurrence of the amorphous nature of spray-dried powder caused hygroscopicity, stickiness, and the formation of agglomerates during storage. The amorphous powder contained sugar transformed into a crystalline structure due to rapid weight gain, causing the collapse of microcapsules and triggering instability [56]. The spray-dried EDP with MD and WPC was over 78% amorphous in nature and revealed a less crystalline structure in nature. Similar results were observed for spray-dried lycopene powder with MD, revealing its amorphous nature due to the formation of not well-defined small peaks and noises at 10° to 25° having a very small crystalline structure due to the type of wall material [57]. A similar observation was made for the encapsulation of rosemary oil spray drying with blends of whey protein, which revealed much of the amorphous nature of the powder [58]. The nature of EDP belongs to the amorphous type due to the presence of MD and WPC as carrier agents. The interaction of diosgenin with both carrier agents revealed good binding properties during the feed preparation and spray drying processes.

## 4. Conclusions

The production of the encapsulated diosgenin powder using MD and WPC revealed the stability and process optimization parameters obtained during spray drying. IAT, FFR, and CAC significantly affected the response variables, such as yield, encapsulation efficiency, moisture content, antioxidant activity, hygroscopicity, and solubility, of MD-EDP and WPC-EDP. Higher yield, encapsulation, and antioxidant activity of EDP was obtained with WPC in comparison to MD. The impacts of the carrier agent type and their concentration effects was clearly observed in the responses of EDP for MD and WPC, respectively. The micrographs revealed the smooth and wrinkled-wall effects shown by MD and WPC, respectively. The amorphous nature of EDP showed better stability and quality-preservation properties. EDP had a more significant effect, compared to the selected CAC that can propose much more stable food-grade powder properties. Diosgenin can be encapsulated under the optimum conditions, such as IAT 170 °C, FFR 500 mL/h, and CAC 20%, for the most-suited powder characterization, till further processing. EDP has more potential for being incorporated in various food matrices for delivering health benefits in view of benefiting humans.

## Figures and Tables

**Table 1:** Coded and real values for representation of the independent variables in central composite designs for MD and WPC.

	Inlet Air Temperature (IAT, A °C)		Feed Flow Rate (FFR, B mL/h)		Carrier Agent Concentration (CAC, C %)	
Runs	Coded	Real	Coded	Real	Coded	Real
1	−1	150	−1	300	−1	10
2	+1	170	−1	300	−1	10
3	−1	150	+1	500	−1	10
4	+1	170	+1	500	−1	10
5	−1	150	−1	300	+1	20
6	+1	170	−1	300	+1	20
7	−1	150	+1	500	+1	20
8	+1	170	+1	500	+1	20
9	−1.682	143 *	0	400	0	15
10	+1.682	177 *	0	400	0	15
11	0	160	−1.682	232 *	0	15
12	0	160	+1.682	568 *	0	15
13	0	160	0	400	−1.682	6.6
14	0	160	0	400	+1.682	23.4
15	0	160	0	400	0	15
16	0	160	0	400	0	15
17	0	160	0	400	0	15
18	0	160	0	400	0	15
19	0	160	0	400	0	15
20	0	160	0	400	0	15

* Roundoff to nearest digit.

**Table 2:** Representation of the effects of independent variables on the dependent variables of spray-dried diosgenin powder with MD and WPC.

Run				MD						WPC					
	A- IAT (°C)	B- FFR (mL/h)	C- CAC (%)	Yield (%)	EE (%)	MC (%)	AA (%)	HG (%)	SB (%)	Yield (%)	EE (%)	MC (%)	AA (%)	HG (%)	SB (%)
1	150	300	10	50.42	60.52	1.85	37.57	11.84	87.64	49.04	51.27	2.56	37.34	5.92	70.92
2	160	400	15	48.64	60.45	0.65	47.94	11.44	91.12	44.57	60.05	1.45	43.59	6.25	76.96
3	170	500	20	67.75	62.16	0.75	52.71	9.76	94.12	52.34	65.17	2.06	38.44	9.38	82.92
4	160	400	15	69.43	54.29	1.47	37.77	8.04	92.4	53.63	65.18	2.05	38.74	9.15	83.48
5	150	500	10	37.48	58.37	1.75	46.42	11.09	78.88	53.94	64.28	1.95	39.42	9.19	82.32
6	160	400	6.6	50.23	58.79	1.88	39.59	11.78	88.76	53.78	63.35	2.09	39.08	9.6	83.12
7	150	300	20	51.77	58.21	1.74	38.85	11.20	87.44	82.25	88.60	0.98	52.26	4.82	85.81
8	170	300	20	34.12	46.78	2.58	42.76	11.27	71.52	74.53	83.23	0.97	49.28	7.24	88.12
9	170	500	10	80.65	59.91	0.79	51.19	6.64	91.23	50.53	67.78	0.82	48.05	10.53	86.26
10	150	500	20	59.65	50.49	0.97	47.89	11.58	91.6	71.68	63.71	1.60	38.40	6.32	87.18
11	160	568	15	39.76	58.51	2.06	45.41	13.52	86.8	69.46	86.41	0.96	53.95	7.09	89.28
12	160	232	15	51.34	55.29	1.86	38.42	11.68	88.24	59.12	69.11	0.85	44.28	9.69	91.72
13	160	400	15	42.98	83.33	1.85	45.72	14.42	87.48	39.45	58.41	1.98	47.51	8.92	73.8
14	160	400	23.4	51.76	56.47	1.84	37.10	11.34	87.72	53.55	64.61	2.09	38.30	9.37	82.12
15	160	400	15	72.67	53.73	0.75	48.24	9.78	93.12	63.65	70.35	1.79	46.64	6.94	82.42
16	177	400	15	57.33	81.58	0.65	43.63	11.17	96.64	36.76	55.49	2.76	43.21	9.66	65.48
17	143	400	15	47.99	78.08	2.39	36.63	7.65	75.84	52.79	63.12	2.03	39.46	9.22	83.04
18	160	400	15	51.85	67.43	1.87	37.41	11.57	88.36	61.37	72.96	1.17	46.13	9.28	86.88
19	160	400	15	42.74	58.16	1.27	42.97	8.23	81.68	41.87	61.08	2.25	43.59	11.99	81.8
20	170	300	10	61.57	65.19	1.58	45.19	8.75	87.72	44.87	63.78	2.06	46.57	12.64	82.64

MD: maltodextrin; WPC: whey protein concentrate; EE = encapsulation efficiency; MC = moisture content; AA = antioxidant activity; HG = hygroscopicity; SB = solubility; IAT: inlet air temperature; FFR: feed flow rate; and CAC: carrier agent concentration.

**Table 3:** Representation of the significant levels (*p*-values) for the dependent variables of spray-dried diosgenin powder with MD and WPC.

	MD						WPC					
	Yield (%)	EE (%)	MC (%)	AA (%)	HG (%)	SB (%)	Yield (%)	EE (%)	MC (%)	AA (%)	HG (%)	SB (%)
Model	<0.0001	<0.0001	<0.0001	<0.0001	<0.0001	<0.0001	<0.0001	<0.0001	<0.0001	<0.0001	<0.0001	<0.0001
A	<0.0001	<0.0001	<0.0001	<0.0001	<0.0001	<0.0001	<0.0001	<0.0001	<0.0001	<0.0001	<0.0001	<0.0001
B	<0.0001	0.1924	<0.0001	0.0035	<0.0001	<0.0001	<0.0001	0.0882	<0.0001	<0.0001	<0.0001	<0.0001
C	<0.0001	<0.0001	<0.0001	0.0032	<0.0001	<0.0001	<0.0001	<0.0001	<0.0001	<0.0001	<0.0001	<0.0001
AB	0.3030	0.2245	0.9924	0.0655	0.1032	0.0005	0.0206	0.1820	0.0113	<0.0001	<0.0001	<0.0001
BC	0.4099	0.0008	0.0673	0.0023	0.0170	<0.0001	0.1984	0.0333	<0.0001	<0.0001	<0.0001	<0.0001
CA	0.0854	0.0019	<0.0001	0.0002	0.0008	0.0009	<0.0001	<0.0001	<0.0001	<0.0001	<0.0001	0.0007
A^2^	0.0025	0.4475	0.0172	0.0002	<0.0001	<0.0001	0.4944	0.2142	0.7233	0.0002	0.0050	0.0002
B^2^	0.1411	0.1852	<0.0001	<0.0001	0.0016	<0.0001	0.4330	0.0004	0.1746	<0.0001	<0.0001	<0.0001
C^2^	<0.0001	<0.0001	<0.0001	<0.0001	<0.0001	0.0012	0.2823	0.0005	<0.0001	<0.0001	0.0002	0.0003
R^2^	0.9973	0.9848	0.9921	0.9830	0.9847	0.9909	0.9961	0.9874	0.9910	0.9955	0.9906	0.9918
ADJ R^2^	0.9948	0.9711	0.9850	0.9677	0.9710	0.9827	0.9925	0.9760	0.9829	0.9914	0.9821	0.9843
CV	1.64	2.67	4.71	2.09	3.14	0.9328	1.88	2.23	4.45	1.06	3.11	0.9681
Lack of fit	0.244 ^NS^	0.071 ^NS^	0.129 ^NS^	0.616 ^NS^	0.171 ^NS^	0.071 ^NS^	0.063 ^NS^	0.058 ^NS^	0.096 ^NS^	0.606 ^NS^	0.073 ^NS^	0.087 ^NS^

MD: maltodextrin; WPC: whey protein concentrate; EE = encapsulation efficiency; MC = moisture content; AA = antioxidant activity; HG = hygroscopicity; and SB = solubility. NS: non-significant.

**Table 4:** Response variables and their model equations for MD and WPC for EDP.

Response Variables	Response Model Equations for MD as Carrier Agent	Response Model Equations for WPC as Carrier Agent
Yield (%)	+51.23 + 2.97 A − 3.98 B + 13.09 C + 0.3365 AB − 0.2665 AC + 0.5914 BC + 0.9261 A^2^ − 0.3689 B^2^ + 2.78 C^2^	+ 53.32 + 2.96 A – 3.92 B + 13.00 C +0.2168 AB − 0.3017AC + 0.4197 BC + 0.7555 A^2^ − 0.3790 B^2^ + 2.75 C^2^
Encapsulation Efficiency (%)	+59.33 + 6.26 A + 0.6211 B + 7.86 C − 0.7515 AB + 2.74 AC + 2.43 BC − 0.3419 A^2^ -0.6154 B^2^ + 3.97 C^2^	+ 64.25 + 6.25 A − 0.7625 B + 8.06 C − 0.6995 AB + 2.79 AC + 2.63 BC − 0.5636 A^2^ + 0.9693 B^2^ + 3.47 C^2^
Moisture content (%)	+1.84 − 0.3724 A + 0.3549 B − 0.2660 C − 0.0003 AB − 0.0523 AC − 0.3273 BC − 0.0541 A^2^ − 0.2059 B^2^ − 0.1989 C^2^	+ 2.05 − 3610 A + 0.3610 B − 0.2659 C − 0.0099 AB − 0.0396 AC − 0.3224 BC -0.0625 A^2^ − 0.2084 B^2^ − 0.1988 C^2^
Antioxidant activity (%)	+38.17 + 3.26 A + 0.9271 B + 0.9436 C − 0.6605 AB + 1.30 AC + 1.83 BC + 1.39 A^2^ + 2.24 B^2^ + 3.70 C^2^	+ 38.92 + 2.77 A + 0. 8724 B + 1.30 C − 0.9536 AB + 1.67 AC + 2.22 BC + 0.9461 A^2^ + 2.25 B^2^ + 3.83 C^2^
Hygroscopicity (%)	+11.58 + 1.08A + 0.8256 B − 1.14 C − 0.2119 AB − 0.3377 AC − 0.5550 BC − 0.7454 A^2^ + 0.3790 B^2^ − 1.01 C^2^	+ 9.33 + 0.9763 A + 0.7538 B − 1.30 C − 0.3410 AB − 0.6908 AC − 0.5502 BC − 0.6574 A^2^ + 0.6012 B^2^ − 0.9175 C^2^
Solubility (%)	+88.01 + 4.27 A − 2.46 B + 4.17 C + 1.44 AB − 2.20 AC + 1.35 BC − 1.43 A^2^ + 1.52 B^2^ − 0.9598 C^2^	+ 82.82 + 4.41 A − 2.55 B + 4.15 C + 1.62 AB − 2.23 AC + 1.54 BC − 1.33A^2^ + 1.60 B^2^ − 1.01 C^2^

A: inlet air temperature; B: feed flow rate, and C: carrier agent concentration.

**Table 5:** Representation of the upper and lower limits of the process parameters, followed by the predicted and experimental values for the selected process responses for EDP production.

		Upper and Lower Limits of Variables					Predicted and Experimental Values for the Optimized Variable					
		Aim	MD		WPC		MD			WPC		
			Lower Limit	Upper Limit	Lower Limit	Upper Limit	Pred. Value	Exptl. Value	Variation (%)	Pred. Value	Exptl. Value	Variation (%)
Factors	A	In range	150	170	150	170	170			170		
	B	In range	300	500	300	500	500			500		
	C	In range	10	20	10	20	20			20		
Responses	Yield (%)	Max.	34.12	80.65	36.76	82.25	51.23	50.21	1.09	75.35	74.54	1.06
	EE (%)	Max.	46.78	83.33	51.27	88.60	59.33	60.01	1.15	84.05	83.23	0.97
	MC (%)	Min.	0.65	2.58	0.821	2.763	1.84	1.76	4.18	0.88	0.92	4.55
	AA (%)	Max.	36.63	52.71	37.34	53.95	38.17	38.36	0.50	49.55	49.28	0.55
	HG (%)	Min.	6.64	14.42	4.82	12.64	11.57	11.89	2.80	7.48	7.25	3.06
	SB (%)	Max.	71.52	96.64	65.48	91.72	88.01	88.19	0.19	87.81	88.12	0.36

MD: maltodextrin; WPC: whey protein concentrate; EE = encapsulation efficiency; MC = moisture content; AA = antioxidant activity; HG = hygroscopicity; and SB = solubility.

## Data Availability

The data presented in this study are available on request from the corresponding author.
